# Volume-rendered hemorrhage-responsible arteriogram created by 64 multidetector-row CT during aortography: utility for catheterization in transcatheter arterial embolization for acute arterial bleeding

**DOI:** 10.1186/2193-1801-3-67

**Published:** 2014-02-04

**Authors:** Hiroki Minamiguchi, Nobuyuki Kawai, Morio Sato, Akira Ikoma, Hiroki Sanda, Kouhei Nakata, Fumihiro Tanaka, Motoki Nakai, Tetsuo Sonomura, Kazuhiro Murotani, Seiki Hosokawa, Tadayoshi Nishioku

**Affiliations:** Department of Radiology, Wakayama Medical University, 811-1 Kimiidera, Wakayamashi, Wakayama, 641-8510 Japan

**Keywords:** Volume-rendered hemorrhage-responsible arteriogram, CT during aortography, Acute arterial bleeding, Transcatheter arterial embolization, Multidetector-row CT

## Abstract

Aortography for detecting hemorrhage is limited when determining the catheter treatment strategy because the artery responsible for hemorrhage commonly overlaps organs and non-responsible arteries. Selective catheterization of untargeted arteries would result in repeated arteriography, large volumes of contrast medium, and extended time. A volume-rendered hemorrhage-responsible arteriogram created with 64 multidetector-row CT (64MDCT) during aortography (MDCTAo) can be used both for hemorrhage mapping and catheter navigation. The MDCTAo depicted hemorrhage in 61 of 71 cases of suspected acute arterial bleeding treated at our institute in the last 3 years. Complete hemostasis by embolization was achieved in all cases. The hemorrhage-responsible arteriogram was used for navigation during catheterization, thus assisting successful embolization. Hemorrhage was not visualized in the remaining 10 patients, of whom 6 had a pseudoaneurysm in a visceral artery; 1 with urinary bladder bleeding and 1 with chest wall hemorrhage had gaze tamponade; and 1 with urinary bladder hemorrhage and 1 with uterine hemorrhage had spastic arteries. Six patients with pseudoaneurysm underwent preventive embolization and the other 4 patients were managed by watchful observation. MDCTAo has the advantage of depicting the arteries responsible for hemoptysis, whether from the bronchial arteries or other systemic arteries, in a single scan. MDCTAo is particularly useful for identifying the source of acute arterial bleeding in the pancreatic arcade area, which is supplied by both the celiac and superior mesenteric arteries. In a case of pelvic hemorrhage, MDCTAo identified the responsible artery from among numerous overlapping visceral arteries that branched from the internal iliac arteries. In conclusion, a hemorrhage-responsible arteriogram created by 64MDCT immediately before catheterization is useful for deciding the catheter treatment strategy for acute arterial bleeding.

## Introduction

The concept of installing a CT scanner and angiography unit in the same room, utilizing a shared tabletop, originated in Japan for conducting CT during arterial portography (CTAP) and CT hepatic arteriography (CTHA) (Matsui et al. [1985]; Hayashi et al. [2002]; Honda et al. [2000]; Kitao et al. [2009]; Matsui et al. [2011]). The combined facility is termed angio CT or interventional radiology CT (IVRCT). Angio CT is particularly useful for investigating the hemodynamics of small hepatocellular carcinoma (HCC), and has enabled improved understanding of the growth process from early HCC to mature HCC (Matsui et al. [1985]; Hayashi et al. [2002]; Honda et al. [2000]; Kitao et al. [2009]; Matsui et al. [2011]; Lee et al. [2002]). Hepatoma-feeding arteriograms that were created by MDCT during aortography (MDCTAo) were previously reported to be displayed on the monitor in the angiography room and used for catheterization of feeding arteries to a HCC (Minamiguchi et al. [2013]). Accordingly, it should be possible to apply MDCTAo to patients with acute arterial bleeding, to map the hemorrhage site and to navigate catheterization to the artery responsible for hemorrhage.

The purpose of the present paper is to introduce the method for creating a hemorrhage-responsible arteriogram by MDCTAo, and to describe the clinical usefulness of the hemorrhage-responsible arteriogram for catheter treatment of acute arterial bleeding.

## Review

### Interventional radiology multidetector-row CT

We used a 64 multidetector-row CT (64MDCT) scanner installed in an angiography room. The scanner and the angiography suite have a common tabletop, removing the need to transfer the patient between procedures. A 64MDCT scanner (0.5 sec/rotation, pitch factor 1.484; INFX 8000-C Aquilion CX, Toshiba Medical, Tochigi, Japan) with collimation and detector thickness each of 0.5 mm was used for MDCTAo. The arterial scans were reconstructed with slice thickness and slice interval each of 0.5 mm, and maximum intensity projection (MIP) and volume-rendered (VR) images were produced instantly using a three-dimensional (3D) workstation (ZIO Station, ZIO Soft, Tokyo, Japan). Tube voltage was 120 kVp with tube mA determined automatically.

The scanning field is determined to include the hemorrhage and the artery responsible for hemorrhage. The position of the catheter tip and the amount of contrast medium are adjusted according to the scanning field. We routinely use three protocols: for thoracic arterial bleeding, abdominal arterial bleeding, and pelvic arterial bleeding.

### MDCT during thoracic aortography (MDCT-TA) for thoracic arterial bleeding

We developed a scanning protocol for MDCT-TA for hemoptysis based on our clinical experience. Briefly, scans were obtained from the level of the upper margin of the 7^th^ cervical vertebra to the lower margin of the 1^st^ lumbar vertebra. MDCT-TA scanning was scheduled to start with a contrast medium (CM) injection rate of 10 mL/sec, using a 4 F pigtail catheter as used for thoracic aortography (TA; 90 cm, Medikit, Miyazaki, Japan), positioned at the ascending aorta. In TA of each of 10 patients with hemoptysis, a total of 30 mL of CM was injected at a rate of 10 mL/sec, and the mean time from initiation of the CM injection to the end of peak density in the descending aorta at the level of the diaphragm was approximately 5 sec. Therefore, MDCT scanning during TA could be delayed for 5 sec after the initiation of CM injection. The concentration of CM used was 150 mg iodine/ml. We no longer conduct conventional TA for thoracic arterial bleeding since the development of this protocol.

### MDCT during abdominal aortography (MDCT-AA) for abdominal arterial bleeding

The protocol used for MDCT-AA follows that for detecting the feeding artery for hepatocellular carcinoma described in a previous report (Minamiguchi et al. [2013]). In short, a 3 F pigtail catheter (70 cm, Medikit) was advanced so that its tip was positioned at the level of the tracheal bifurcation. Although the scan range varied depending on the suspected site of acute arterial bleeding, scans were mostly obtained from the level of the diaphragm to the level of the aortic bifurcation. A total of 90 ml of 2-fold diluted CM (Iopamidol 300, Bracco, Milano, Italy) was injected via the pigtail catheter at a rate of 9 ml/sec for 10 sec. MDCT scanning was initiated at 7 sec from the beginning of CM injection at maximum expiration.

### MDCT during pelvic arteriography (MDCT-PA) for pelvic arterial bleeding

A pigtail catheter was advanced to the upper level of the renal arteries in order to include the branching of the ovarian or testicular arteries. Scans were obtained from the level superior to the renal arteries to the lower border of the ischium. A total of 80 ml of 2-fold diluted CM (Iopamidol 300) was injected via the pigtail catheter at a rate of 8 ml/sec for 10 sec. MDCT scanning was initiated at 7 sec from the beginning of CM injection at maximum expiration.

### Creation of hemorrhage-responsible arteriogram

The hemorrhage-responsible arteriogram is created on the same basis as that reported for a hepatoma-feeding arteriogram (Minamiguchi et al. [2013]). In short, the hemorrhage-responsible arteriogram using MDCTAo is synthesized using three basic VR images: a background bone VR image, an aorta–branch artery VR image, and a hemorrhage–responsible artery VR image. The background bone VR image is produced by deleting all components except the vertebrae and dorsal ribs from the axial MIP image. The aorta–branch artery VR image is produced by deleting all of the bone and cartilage components from the axial MIP image, leaving the aorta, visceral arteries, and visceral organs.

The hemorrhage-responsible artery VR image is produced by marking the hemorrhage, pseudoaneurysm and/or new vessels, and then identifying the responsible arteries. After identification, extension software (ZIO station) automatically connects the responsible artery to the branch artery arising from the aorta, to create a hemorrhage-responsible artery VR image.

In MDCT-TA for hemoptysis, the hemoptysis-responsible VR image was produced by following the path of newly dilated vessels surrounding the bronchus and/or stain corresponding to the inflammation or bleeding. The image was then extended to the aorta–branch artery VR image.

In MDCT-AA for acute abdominal artery bleeding, extravasation of contrast medium and the surrounding area was first detected, the artery responsible for extravasation was identified, and a hemorrhage-responsible artery VR image was then created.

In MDCT-PA for pelvic artery bleeding, we used the subtraction method to obtain a background bone VR image and an aorta to branch artery VR image because this region is relatively unaffected by respiration movement. Specifically, unenhanced CT and MDCT-PA were obtained sequentially in approximately 15 sec during the same breath-hold. A background bone VR image was obtained from the unenhanced CT, and an aorta–branch artery VR image was instantly obtained by subtracting the entire unenhanced CT images from the entire MDCT-PA images. This subtraction method accelerated the production process. A hemorrhage-responsible VR image was produced using the same method as that described above, and extended to produce an aorta–branch artery VR image.

### Application of CT during aortography for acute arterial bleeding

In the last 3 years at our institute, 71 patients suspected of having acute arterial bleeding underwent MDCTAo, using MDCT installed in the angiography room. Because most patients were in a critical state of shock, receiving continuous blood transfusion and requiring emergent hemostasis by embolization, CT angiography via the venous route was not always performed. Most patients retained consciousness and could perform breath holding. We generally did not conduct MDCTAo in unresponsive patients. However, when we did perform MDCTAo on unconscious, ventilated patients, the respirator was used to perform breath holding. No patients with pelvic fractures were included in this series, except one patient who had multiple fractures of bilateral iliac, ischial and pubic bones, causing hemorrhagic shock. Of the 71 patients, 69 received MDCTAo, and 2 received CT during superior mesenteric arteriography because the responsible arteries were already known to branch from the superior mesenteric artery. The origins of the acute arterial bleeding in the 71 patients were as follows: hemoptysis (n = 27); hemothorax (n = 2); bleeding post-pancreatectomy (n = 12); intraperitoneal bleeding (n = 3); gastrointestinal bleeding (n = 6); retroperitoneal bleeding (n = 9); uterine bleeding (n = 3); bleeding into the urinary bladder, (n = 3); pelvic fracture (n = 1); intra-pelvic hematoma (n = 1); and subcutaneous hematoma on the trunk (n = 4).

The arteries responsible for hemorrhage were visualized and treated in 61 of the 71 patients. The embolic materials used were gelatin sponge particles (GSP) in 38 patients, n-butyl cyanoacrylate (NBCA) in 10 patients, NBCA plus microcoils in 1 patient, and microcoils in 9 patients. Of 12 patients with bleeding post-pancreatectomy, 3 cases of pseudoaneurysm at the superior mesenteric artery trunk were treated by stent-graft. Hemorrhage was not visualized in the remaining 10 patients, of whom 6 had a pseudoaneurysm in a visceral artery; 1 with urinary bladder bleeding and 1 with chest wall hemorrhage had gaze tamponade; and 1 with urinary bladder hemorrhage and 1 with uterine hemorrhage had spastic arteries. Six patients with pseudoaneurysm underwent preventive embolization and the other 4 patients were managed by watchful observation.

### Advantages of hemorrhage-responsible arteriogram in catheterization for acute arterial bleeding

**Figure 1:**
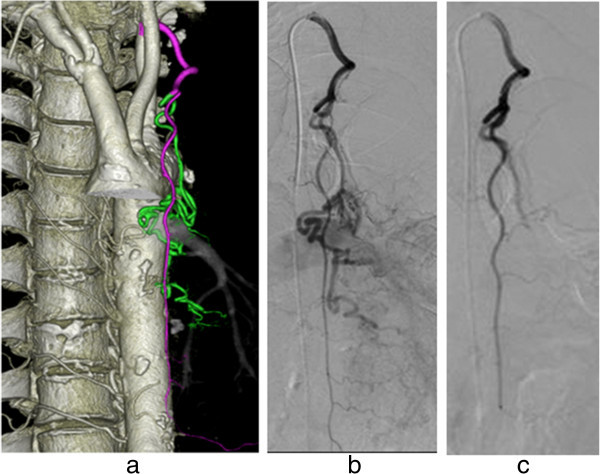
**A case of hemoptysis.** Hemorrhage-responsible arteriogram created by CT during thoracic aortography depicts the left bronchial artery (green) branching from the left intra-thoracic artery (purple) **(a)**. A microcatheter was advanced via a preshaped catheter and inserted into the left intra-thoracic artery, followed by selective embolization of the left bronchial artery with gelatin sponge particles as shown in the angiography before **(b)** and after **(c)** embolization.

**Figure 2:**
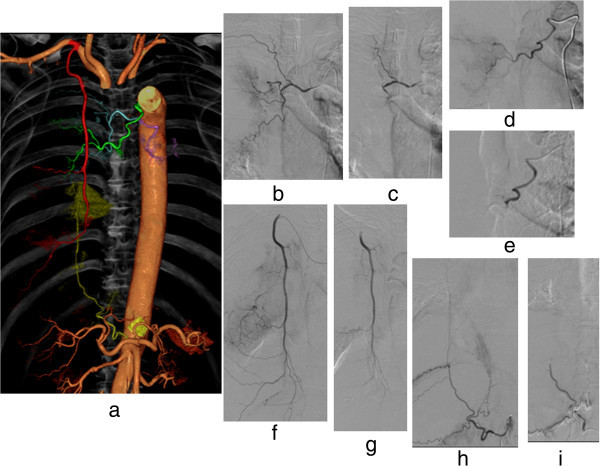
**A case of hemoptysis. (a)** Hemorrhage-responsible arteriogram created by CT during thoracic aortography depicts the arteries responsible for hemoptysis: the right bronchial artery branching from the thoracic aorta (blue); the right bronchial artery branching from the aortic arch (green); the right inferior phrenic artery branching from the celiac axis (yellow); and the right intrathoracic artery branching from the right subclavian artery (red). Based on this image, embolization with gelatin sponge particles was conducted of each of these arteries: the right bronchial artery branching from the thoracic aorta **(b, c)**; another bronchial artery branching from the aortic arch **(d, e)**; the right intra-thoracic artery branching from the right subclavian artery **(f, g)**; and the right inferior phrenic artery branching from the celiac axis artery **(h, i)**.

Rapid hemostasis is essential in emergent acute arterial bleeding (Yonemitsu et al. [2009]). It is desirable to detect the artery responsible for hemorrhage quickly, using the smallest possible volume of CM. It is difficult to compare CT angiography and MDCTAo in terms of their ability to detect hemorrhage because most patients in the present study did not receive CT angiography via the venous route. CTAo was conducted to investigate both the extravasation and the responsible artery for hemorrhage. Conventionally, the artery suspected to be responsible for hemorrhage is catheterized after aortography; however, it is sometimes difficult to detect the responsible artery for hemorrhage because of overlapping of the vessels. If selective arteriography reveals negative findings for hemorrhage for the suspected artery, then another artery is catheterized, followed by repeated angiography. This process is time consuming, requires a large volume of CM, and places a high radiation burden on the patient. In contrast, the single scan obtained with MDCTAo enables investigation of all arteries, even if multiple arteries are responsible for hemorrhage, and can demonstrate whether the bronchial arteries or arteries from another system (e.g., the internal thoracic, lateral thoracic, or inferior phrenic arteries) are responsible for hemoptysis (Figures 1 and 2) (Ziyawudong et al. [2012]; Hartmann et al. [2007]; Bruzzi et al. [2006]; Remy-Jardin et al. [2004]).

**Figure 3:**
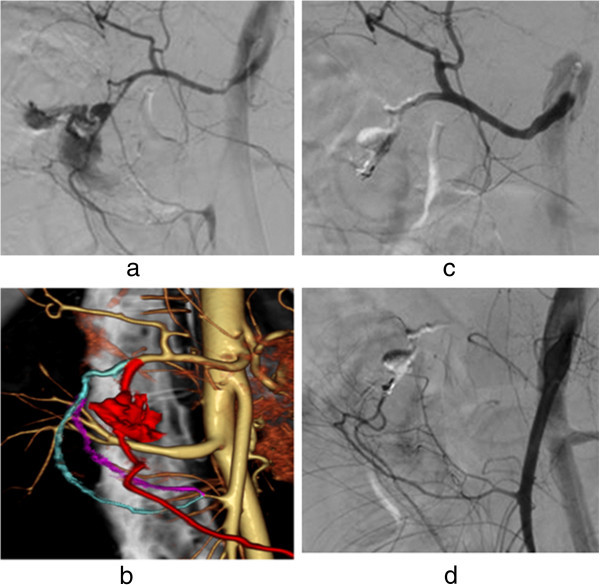
**A case of bleeding from duodenal ulcer. (a)** Celiac arteriography depicted the overlapping of the extravasation of contrast medium, gastroduodenal artery (GD), posterior pancreatico-duodenal artery (P-PDA) and anterior pancreatico-duodenal artery (A-PDA). **(b)** Hemorrhage-responsible arteriogram created by CT during abdominal aortography depicted that GD (red) was the responsible artery to hemorrhage but not P-PDA (purple) and A-PDA (blue). **(c)** Celiac arteriography following embolization of with n-butyl cyanoacrylate and microcoils depicted no extravasation of contrast medium. **(d)** Superior mesenteric arteriography depicted no extravasation of contrast medium.

**Figure 4:**
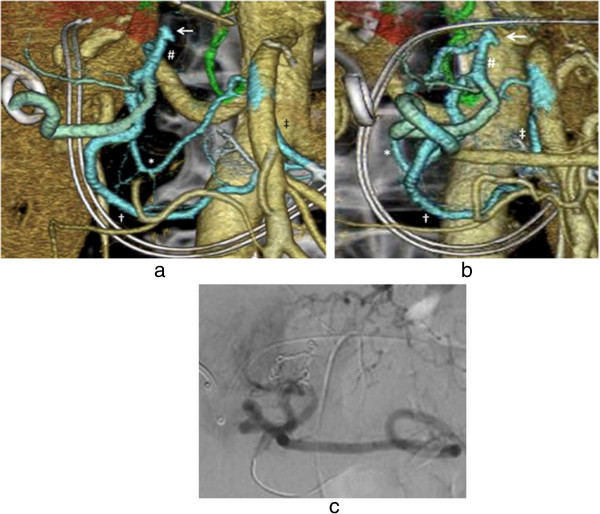
**A case of bleeding post-pancreatectomy following Appleby operation for pancreatic body cancer with ligation of the common hepatic artery.** A hemorrhage-responsible arteriogram created by CT during abdominal aortography depicts the pseudoaneurysm (arrow) situated at the end of the gastroduodenal artery (#) on the anterior–posterior view **(a)**; the right anterior oblique view **(b)** separates the overlapping vessels, enabling microcoil embolization from the posterior superior pancreaticoduodenal artery (*) to the gastroduodenal artery (#) via the anterior superior pancreaticoduodenal (†) arcade, through the anterior inferior pancreaticoduodenal artery (‡) branching from the superior mesenteric artery. Based on the right anterior oblique view, microcoil embolization was conducted **(c)**.

**Figure 5:**
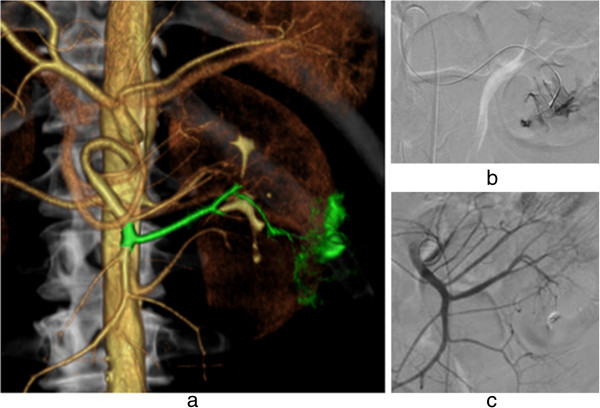
**A case of endoscopically unidentified intestinal bleeding. (a)** Hemorrhage-responsible arteriogram created by CT during abdominal aortography depicts the jejunal artery (green) as the source of the bleeding. Using this image, the jejunal branch artery was catheterized using a microcatheter **(b)**, followed by embolization with n-butyl cyanoacrylate **(c)**.

**Figure 6:**
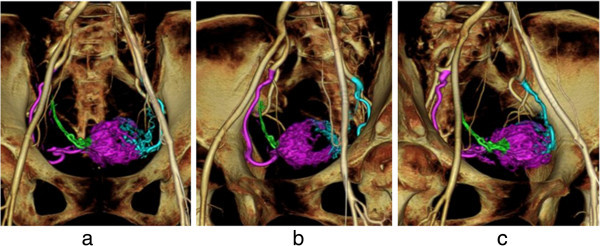
**A case of uterine bleeding following surgical removal of a destructive mole.** Hemorrhage-responsible arteriogram (HRA) created by CT during pelvic arteriography depicts the right (purple color) and left (blue color) uterine arteries **(a)**. The left anterior oblique HRA image enabled easy insertion of the catheter into the right uterine artery (purple color) **(b)**, while the right anterior oblique view enabled clear visualization of the left uterine artery (blue color) **(c)**. Only the right uterine artery was embolized (using gelatin sponge particles) to retain the patient’s ability to bear children in the future.

**Figure 7:**
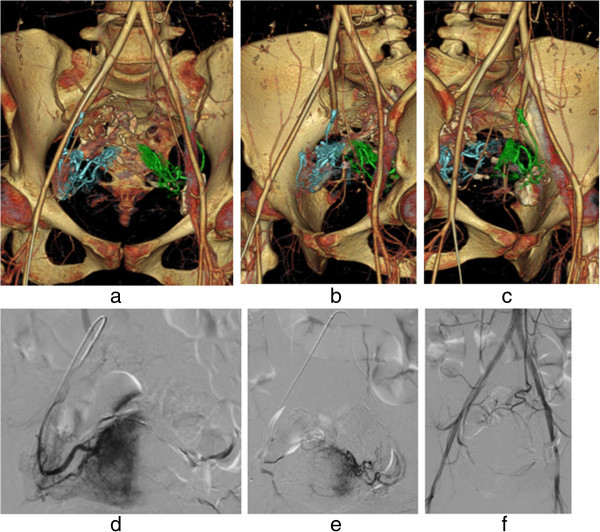
**A case of uterine bleeding caused by a uterine cervix pregnancy at 5 weeks.** Hemorrhage-responsible arteriogram (HRA) created by CT during pelvic arteriography depicts the bilateral uterine arteries responsible for uterine bleeding **(a)**. Using oblique HRA images, the right uterine artery (blue color) **(b)** and the left uterine artery (green color) **(c)** were selectively catheterized. Angiographic images of each of the bilateral uterine arteries depict a hypervascular stain corresponding to the placenta **(d, e)**. The patient received actinomycin D infusion for abortion followed by embolization with gelatin sponge particles **(f)**.

MDCTAo is especially useful for detecting hemorrhage in the region of the pancreatic head and for identifying arterial supply from the celiac artery and/or superior mesenteric artery (Figures 3 and 4) (Izaki et al. [2011]). Furthermore, although overlapping with other contrast-stained organs causes difficulties in identifying extravasation of CM in angiography, MDCTAo resolves this problem (Figures 3, 4 and 5). In a case of pelvic hemorrhage in which the artery responsible for hemorrhage had to be identified from among several visceral arteries branching from internal iliac arteries, MDCTAo could identify and map the artery responsible for hemorrhage. In navigation for catheterization, the image could be rotated to the most helpful view for catheter insertion (Figures 6 and 7). These examples show that interventional radiologists can use a hemorrhage-responsible arteriogram in various situations to navigate to the target responsible artery. MDCTAo accelerates the process of deciding the catheter treatment strategy at the point of investigating the responsible artery, because the hemorrhage-responsible arteriogram enables identification of the responsible artery and its connection to the aortic branch artery. MDCTAo with 64MDCT can improve speed and reliability in treatment of acute arterial bleeding.

### Limitations

It takes 5–10 min to create a hemorrhage-responsible arteriogram, which is a long time in the situation of acute arterial bleeding. Although this period can be used to exchange the pigtail catheter for the pre-shaped catheter, insert the microcatheter, and bend the micro-guidewire tip, it would be ideal to view the hemorrhage-responsible arteriogram on the display without delay. This could be achieved by development of software that could create a catheter navigation image within a few minutes. Another limitation is that MDCTAo cannot depict hemorrhage when acute arterial bleeding has temporarily ceased. Furthermore, when MDCTAo depicts hemorrhage in the small intestine, the hemorrhage-responsible arteriogram does not reflect the true hemorrhage site for catheterization if the small intestine has moved after scanning, as is common.

## Conclusions

In conclusion, although having limitations, we found that a hemorrhage-responsible arteriogram created by 64MDCT immediately before catheterization is useful for devising the catheter treatment strategy in acute arterial bleeding.

## Authors’ information

Article notes: The contents of this manuscript were presented as a luncheon seminar at the International Abdominal Radiological Meeting, 2013, held in Taiwan, and as a special lecture at the 2013 New Technology International Forum of Interventional Radiology and Imaging Diagnosis, held in NanChon, China.
